# Rational Design of Mesoporous Silica (SBA-15)/PF (Phenolic Resin) Nanocomposites by Tuning the Pore Sizes of Mesoporous Silica

**DOI:** 10.3390/ma15248879

**Published:** 2022-12-12

**Authors:** Hongxia Liu, Yijia Lao, Jiayi Wang, Junjie Jiang, Chuanbai Yu, Yuanli Liu

**Affiliations:** Guangxi Key Laboratory of Optical and Electronic Materials and Devices, College of Materials Science and Engineering, Guilin University of Technology, Guilin 541004, China

**Keywords:** SBA-15/PF, nanocomposites, pore expanding, thermal properties, frictional properties

## Abstract

The development of composite materials with functional additives proved to be an effective way to improve or supplement the required properties of polymers. Herein, mesoporous silica (SBA-15) with different pore sizes were used as functional additives to prepare SBA-15/PF (phenolic resin) nanocomposites, which were prepared by in situ polymerization and then, compression molding. The physical properties and structural parameters of SBA-15 with different pore sizes were characterized by N_2_ adsorption–desorption, X-ray diffraction (XRD), and scanning electron microscopy (SEM). The thermal properties of the SBA-15/PF hybrid were investigated by differential scanning calorimetry (DSC) and thermal gravimetric analysis (TGA). The mechanical, friction, and dynamic mechanical properties of SBA-15/PF nanocomposites were also studied. The results revealed that the pore sizes of SBA-15 have a significant effect on the resulting SBA-15/PF hybrid and SBA-15/PF nanocomposites. The thermal stability of the SBA-15/PF hybrid was dramatically improved in comparison with pure PF. The friction and dynamic mechanical properties of the SBA-15/PF nanocomposites were enhanced significantly. Specifically, the glass transition temperature (Tg) of the nanocomposite increased by 19.0 °C for the SBA-15/PF nanocomposites modified with SBA-15-3. In addition, the nanocomposite exhibited a more stable friction coefficient and a lower wear rate at a high temperature. The enhancement in thermal and frictional properties for the nanocomposites is ascribed to the confinement of the PF chains or chain segments in the mesopores channels.

## 1. Introduction

Polymer nanocomposites have received considerable attention over the past few decades due to their unique characteristics, such as low cost, lightness, flexibility, and process ability, etc. In addition, polymer nanocomposites possess reinforced mechanical, thermal, electrical, optical, magnetic properties, and fire retardancy [1,2,3]. The enhancement of these properties depend on many factors, including category, structures, size, morphology, surface treatment, and dispersion [3,4,5,6,7], etc. There are a few typical nanomaterials used to reinforce polymer composites, such as SiO_2_ [8], layered silicate [6,9], Al_2_O_3_ [10], carbon nanotubes [11], and graphene [12,13], etc.

Mesoporous materials have received considerable interest in various applications, such as catalysts, adsorbents, optic optical/electronic devices, and chromatographic materials due to their diverse pore structures, tunable pore sizes, and large pore volume, which conferred the possibility of confining various organic materials into the mesoporous channels [14,15,16,17]. Moreover, relevant literature has indicated that the mesoporous materials, especially mesoporous silica, could be a kind of promising novel reinforcing additive for polymer composites. Using suitable preparation methods, it is possible to introduce polymer chains or chain segments in mesoporous channels, which make both the mesoporous materials and polymer chains mutually interpenetrate and then, form into an “interpenetrating organic-inorganic network structure” [18,19,20,21]. The structure can improve the interfacial compatibility of the polymer matrix and mesoporous materials, and confine the movement of polymer chains in the composite system at an elevated temperature; this is expected to enhance the integrated performance of the composite materials.

Mesoporous materials have structural advantages in contrast to conventional nanomaterials because of their tunable pore sizes and ordered pore structure; therefore, investigations regarding how to take advantages of most of the structural features of mesoporous materials have been extensively sought. Typically, compatibility and interaction between the polymer matrix and mesoporous materials are the two crucial factors in the properties of polymer/mesoporous nanocomposites; these can be ascribed to the introduction of more polymer chains or chain segments into the pore channels of mesoporous materials, which, in turn, improves the mechanical and thermal properties of polymer nanocomposites [22,23,24,25,26,27,28,29]. Moreover, the introduction of polymer chains or chain segments into the pore channels of mesoporous materials may result in a low dielectric property [30,31,32,33,34,35], a low thermal expansion property [36,37,38], etc. Apparently, the pore size and pore volume of mesoporous materials play a vital role in the process of confining polymer chains into pore channels. Nevertheless, to the best of our knowledge, investigation of the effect of different pore size and pore volume of mesoporous material on the properties of polymer/mesoporous nanocomposites is still rare.

In the present study, several different pore sizes of SBA-15 were prepared by adding pore expanding agents; for simplicity, the resultant SBA-15 are denoted as SBA-15-n, where the n (1, 2, 3, 4, 5) represents SBA-15 with a different pore size. The effect of the change in structure parameters of SBA-15 on the properties of the resulting SBA-15-n/PF hybrid were systematically investigated. Mesoporous materials, SBA-15, and its pore expanding samples, were initially prepared with the sol–gel method; the mesoporous silica were dispersed in a mixture of formaldehyde and phenol by ultrasonic and subsequent mechanical stirring. SBA-15/PF hybrids were fabricated by in situ condensation polymerization; then, the nanocomposites that used the SBA-15/PF hybrid as a matrix were fabricated by the compression molding method. The structure and physical properties of SBA-15 and its pore-expanding samples were characterized; and the effects of the pore sizes and pore volumes of SBA-15 on the mechanical, thermal, dynamic mechanical, and frictional properties of the SBA-15/PF nanocomposites were studied, respectively.

## 2. Materials and Methods

### 2.1. Materials

EO_20_PO_70_EO_20_ (Pluronic P123) was purchased from Sigma-Aldrich Co., Ltd. (St. Louis, MO, USA), 1,3,5-trimethyl benzene and tetraethoxysilane (TEOS) silica sources were purchased from Aladdin Chemistry Co., Ltd. (Shanghai, China). Hexamethylenetetramine (HMTA), phenol, and formaldehydeand filler were obtained from Xilong Chemical Co., Ltd. (Shantou, China). The catalytic agent oxalic acid was obtained from Shanghai Chemical Reagent Company. All the chemicals were of reagent-grade and used without being further purified.

### 2.2. Synthesis of Mesoporous Silica Materials

Mesoporous silica, SBA-15 with different average pore sizes were synthesized by templating with the EO_20_PO_70_EO_20_ triblock copolymers and expanding with 1,3,5-trimethyl benzene, via a sol–gel process based on a previous report [14]. In a typical SBA-1 synthesis, 2.0 g of Pluronic P123, X g 1,3,5-trimethyl benzene (X = 0, 0.5, 1.0, 1.5 and 2.0) and concentrated HCl solution (37 wt %, 10 mL) were dissolved in deionized water (80 g) and stirred at 35 °C for 6 h. Then, 4.2 g of TEOS was drop-wisely added into the homogenous solution and stirred at 35 °C for 24 h. Thereafter, the solution was moved to an autoclave and hydrothermally treated for 24 h in a 100 °C oven. The obtained particles were collected after filtration and washed with deionized water 3 times; then, dried at 60 °C in the air; the obtained products were calcined at 550 °C for 6 h. These samples were denoted SBA-15, SBA-15-1, SBA-15-2, SBA-15-3, and SBA-15-4.

### 2.3. Fabrication of SBA-15-n/PF Hybrid

The SBA-15/PF hybrid was prepared through in situ polymerization by adding 3.0 wt % mesoporous silica. The calculated amount of mesoporous silica (SBA-15, SBA-15-1, SBA-15-2, SBA-15-3, or SBA-15-4), 30.0 g of phenol and 24.0 mL of formaldehyde (in 37 wt. % water) (P:F molar ratio = 1.15:1), and oxalic acid (2 wt. % of phenol) were added into a three-necked flask with ultrasonic agitation for 30 min. The flask with mixture was then stirred at 85 °C in a water bath for 4 h. The reaction was maintained to remove the water and free phenol at 160–180 °C for an extra 2 h under 0.03–0.05 MPa of pressure, and the final yield of these hybrids was more than 85%. These hybrids were denoted SBA-15/PF, SBA-15-1/PF, SBA-15-2/PF, SBA-15-3/PF, and SBA-15-4/PF.

### 2.4. Preparation of SBA-15-n/PF Nanocomposites

**Figure 1:**
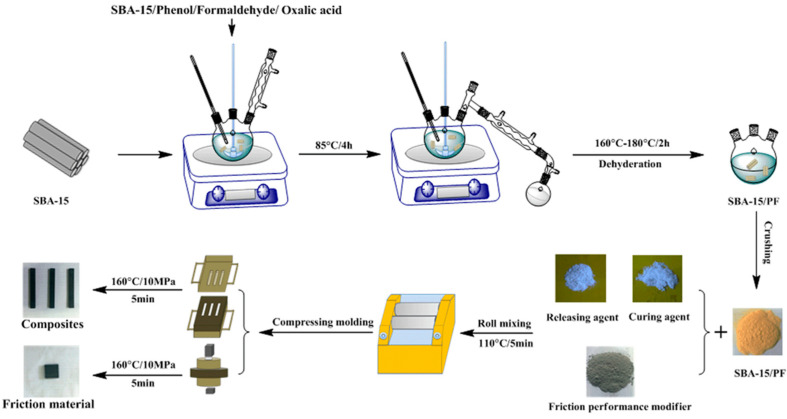
Schematic illustration of fabrication process of the SBA-15/PF hybrid and SBA-15/PF nanocomposites.

The SBA-15-n/PF hybrids with fillers were blended on a roll machine; the mixture was then smashed, and SBA-15-n/PF nanocomposites were prepared by compression molding at 165 °C and 15 MPa for 5 min. Afterward, the composites were postured at 140, 160, and 180 °C for 3 h, respectively. Figure 1 illustrates the experiment details of the fabrication process of the SBA-15/PF hybrid and SBA-15/PF nanocomposites.

### 2.5. Measurements

X-ray powder diffraction (XRD) was performed on a PANalytical X’Pert PRO X-ray diffractometer. The X-ray beam was nickel-filtered with Cu-Kα radiation (λ = 0.154 nm); the diffraction patterns were collected in the 2θ range 0.5–8.0° with a scanning rate of 0.2°/min. N_2_ adsorption–desorption isotherms were accomplished at 77 K using a Quantachrome NOVA 1200e gas-adsorption analyzer. Before the adsorption measurements, all the samples were outgassed at 353 K in the adsorption analyzer degas port for 12 h. The average pore radius and pore volume were determined by the BJH method. The specific surface area was determined using the BET model. The scanning electron microscopy (SEM) measurement was carried out with a S-4800 microscope (Hitachi, Ltd., Tokyo, Japan) operating at 5 kV. The samples were sputter-coated with a thin gold layer under a vacuum situation.

Differential scanning calorimetry (DSC) was conducted with NETZSCH DSC 204 between 10 and 120 °C with a heating rate of 10 °C/min under a nitrogen atmosphere. Thermogravimetric analysis (TGA) was carried out on a NETZSCH STA 449C analyzer operating from 50 to 800 °C with a heating rate of 10 °C/min under a nitrogen atmosphere.

The impact strength of the samples was carried out on a impactor of type JC-25, according to the National Standard of China (GB1843-2008). The specimen was trimmed into a dimension of 120 mm × 10 mm × 4 mm. The flexural properties of the samples were tested on an electronic universal testing machine of type AG-20I, according to the National Standard of China (GB/T9341-2008). The specimen was trimmed into a dimension of 120 mm × 10 mm × 4 mm.

Dynamic mechanical analysis (DMA) was conducted by using a TA Q800 dynamic mechanical analyzer to evaluate the composites’ storage and loss moduli at a fixed frequency of 1 Hz. The heating rate was set as 5 °C/min over the range from 50 to 300 °C. The friction test performed on a constant speed (D-SM) tester; the friction disk was made of cast iron (HT250) with a hardness of 210 HB; the tester provided a friction temperature range of 100–300 °C, which was adjusted automatically; the load was set as 0.98 MPa on each slider, and the speeds were in the interval of 480 r/min. The friction tests were carried out at 100, 150, 200, 250, and 300 °C, respectively, and each test lasted 10 min.

## 3. Results and Discussion

### 3.1. Structure and Properties of Mesoporous Silica SBA-15

**Figure 2:**
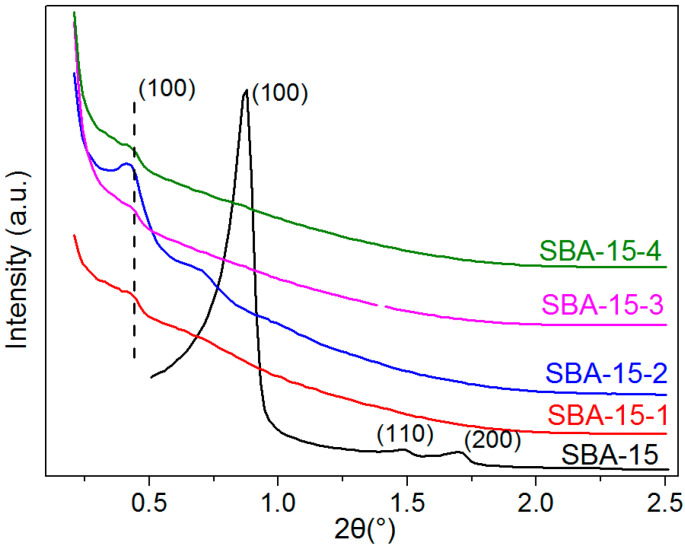
Powder X-ray diffraction (XRD) patterns of SBA-15 particles with different pore sizes.

Five kinds of mesoporous silica, SBA-15 with different average pore sizes were synthesized by templating with the EO_20_PO_70_EO_20_ triblock copolymers and using 1,3,5-trimethyl benzene as a pore-expanding agent via a sol–gel process. Figure 2 shows the XRD patterns of SBA-15 with different average pore sizes. There are three well-resolved peaks at 2θ values between 0.5 and 2.5°, indexed as (100), (110), and (200) Bragg reflections, which can be observed in the X-ray diffraction patterns of SBA-15; this is consistent with a previous report [14]. However, according to the intensity of the peaks at 2θ values less than 0.5°, it can be inferred that the ordering of mesoporous silica with expanded pore size was distinctly weakened. Meanwhile, the XRD peaks of mesoporous silica were shifted to a low angle, which can be ascribed to the increase in pore sizes of the mesoporous silica in view of Bragg’s law about the corresponding relationship between the unit cell parameter and position of the diffraction peak.

**Figure 3:**
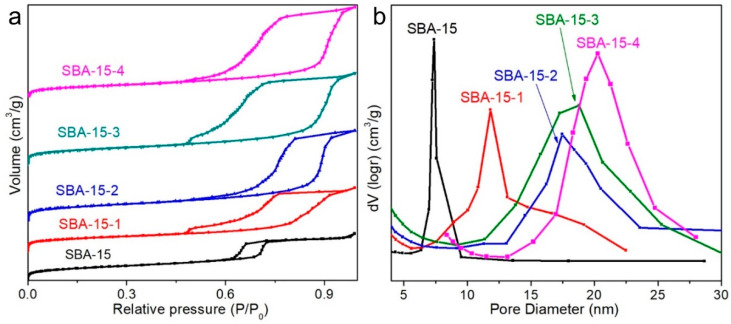
Nitrogen sorption isotherms (**a**) and pore size distribution (**b**) of SBA-15 particles with different pore sizes.

The nitrogen sorption isotherms along with the distribution curves of pore size for the calcined SBA-15 with different average pore sizes are illustrated in Figure 3. The corresponding pore characters, including BET surface areas, total pore volume, and average pore diameters were listed in Table 1. As can be seen from Figure 3a, it is similar to SBA-15, with different pore sizes possessing the typical Langmuir type-IV isotherms with a H_1_ hysteresis loop representing distinct capillary condensation steps, indicating relatively narrow mesoporous size distributions. However, the isotherms of pore-expanding SBA-15 featured capillary condensation steps at a wider pressure range (between 0.45 and 0.95) than that of SBA-15 (between 0.60 and 0.75), manifesting that the mesoporous size distributions of the pore-expanding SBA-15 were wider than that of the original SBA-15. The pore size distributions (PSDs) shown in Figure 3b further suggested that the pore sizes of SBA-15 increased gradually and the pore size distributions widened with the increase of pore-expanding agents. Meanwhile, the BET surface areas of SBA-15 had little change; however, the average pore diameters and total pore volume significantly increased from SBA-15 (7.31 nm and 1.03 cm^3^/g) to SBA-15-4 (20.29 nm and 2.34 cm^3^/g) in Table 1.

**Figure 4:**
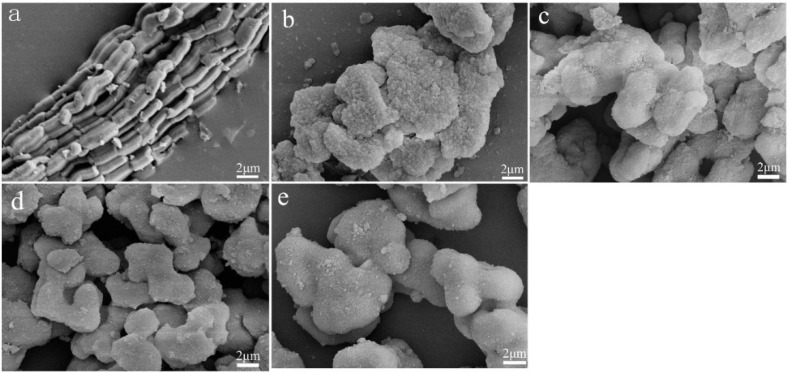
SEM images of calcined SBA-15 (**a**), SBA-15-1 (**b**), SBA-15-2 (**c**), SBA-15-3 (**d**), and SBA-15-4 (**e**).

Figure 4 shows the SEM micrographs of the SBA-15 and SBA-15-3; this clearly shows that the SBA-15 consist of many worm-like shapes with relatively uniform sizes (1–2 μm), which are aggregated together to form clusters. The morphology of SBA-15 is notably inconsistent with a previous report [14]. After pore expanding, SBA-15-3 showed the agglomerated structure, which formed from irregular spherical particles morphology with diameters of 2 μm. It is suggested that the template structure by self-assembly was obviously changed due to adding the pore-expanding agents in the reaction system. Therefore, in relation to its pore-expanding size, SBA-15 transforms shape from a worm-like structure to one that is irregular and spherical.

### 3.2. Thermal Properties of M-SBA-15/PF Hybrid

**Figure 5:**
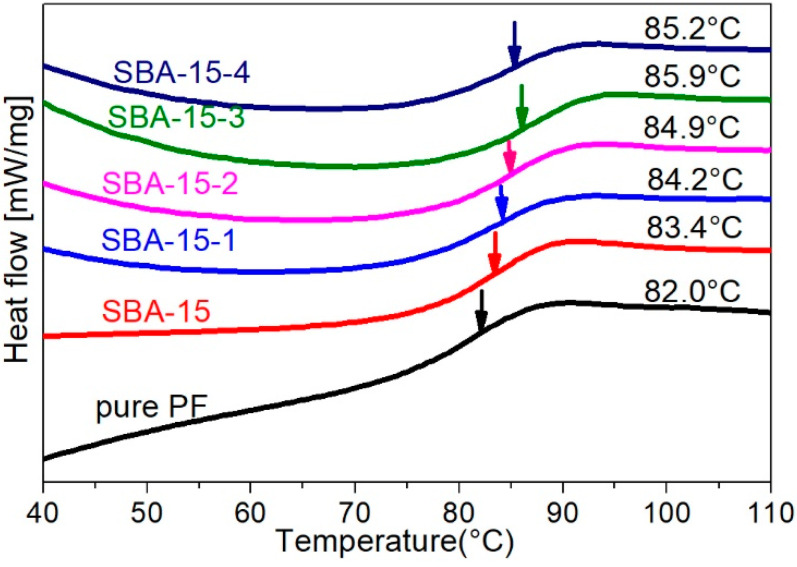
DSC curves and T_g_ values for pure PF and SBA-15/PF hybrid.

Figure 5 shows the DSC curves of pure PF and the SBA-15/PF hybrid. The T_g_ (glass-transition temperature) of pure PF is 82.0 °C; obviously, the SBA-15/PF hybrid shows a higher T_g_ than pure PF. In addition, the T_g_ values of the hybrid increased with the increase in pore size of SBA-15. The enhancement of T_g_ is ascribed to the interaction between SBA-15 with a different pore size and PF molecular chains; this indicated that the existence of SBA-15 with a different pore size may hinder the thermal motions of polymer chains in the hybrid. Nevertheless, its T_g_ value declined slightly for the SBA-15-4/PF hybrid material compared to SBA-15-3/PF; this might be because of the larger surface area and pore volume of SBA-15-3 than SBA-15-4, as indicated in Table 1. Thus, the interaction between the mesoporous silica and PF chains in SBA-15-3/PF was stronger than in SBA-15-4/PF. The T_g_ for the SBA-15/PF hybrid increased only 1.4–3.9 °C, compared to pure PF; the result may be attributed to the strong interaction among PF chains in pure PF of itself; thus, it is limited to enhance the interaction among the PF chains by adding SBA-15 of different pore size in the hybrid.

**Figure 6:**
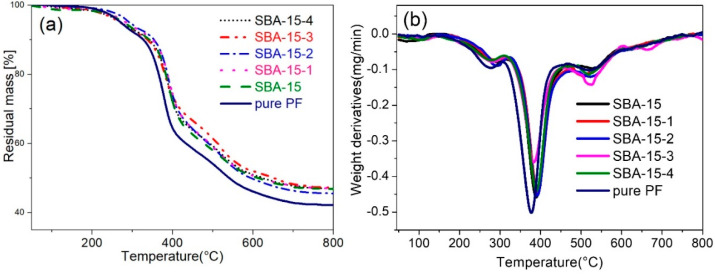
TGA (**a**) and DTG (**b**) curves for pure PF and SBA-15/PF hybrid.

Figure 6 shows the TGA of pure PF and the SBA-15/PF hybrid within the range of 50–800 °C. The thermal stabilities of the hybrid were expressed with a 10% weight loss temperature and maximum thermal decomposing temperatures, namely T_d,10_ and T_d,max_, respectively. As expected, the thermal stabilities of the SBA-15/PF hybrid are higher than pure PF, as shown in Table 2; T_d,10_ and T_d,max_ of all the SBA-15/PF hybrids are increased. For instance, the T_d,10_ and T_d,max_ values for the SBA-15-2/PF hybrid with the addition of 3 wt % increased by 28.7 and 15 °C, respectively, in comparison to pure PF. The char yields are increased by introducing the different pore sizes of SBA-15, which of the SBA-15-3/PF hybrids, was the highest of all. Moreover, as can be seen from Figure 6b, the corresponding derivative curves show the weak peak from 500 to 550 °C, which are more obvious for the hybrid enhanced by the pore-expanding samples. The confinement of polymer chains or chain segments in the mesoporous channels of mesoporous silica undoubtedly play a major role in the improvement of thermal stability properties; and the larger pore size and pore volume that SBA-15 possess, the more the PF chains or chain segments that may be confined into the mesoporous channels of SBA-15 and the more possibility that the thermal stability properties of the hybrid improve.

### 3.3. Mechanical Analysis

**Figure 7:**
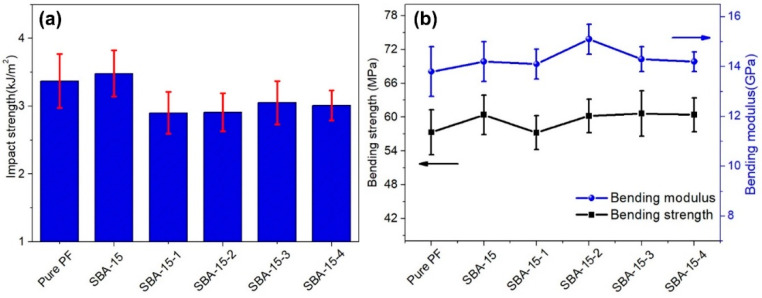
Effects of mesoporous size of SBA-15 on the impact strength of PF nanocomposites (**a**) and bending properties of PF nanocomposites (**b**).

PF nanocomposites were prepared by compression molding using the SBA-15/PF hybrid as a polymer matrix. Mechanical properties of the PF nanocomposites are shown in Figure 7a,b. The impact strength of the PF nanocomposites has obviously not enhanced with the increasing pore size of SBA-15, which was increased for the original SBA-15; however, it was even decreased for the pore-expanding samples. The bending strength and modulus showed a slight enhancement by introducing SBA-15; however, it was difficult to find the correlation between the enhancement and pore size of SBA-15. Thus, the improvement of the mechanical properties was more obvious by introducing the original SBA-15 than the pore-expanding samples. This might be about the shape. The original SBA-15 was similar to fibrous; however, it changed to globular after the pore expansion, as shown in Figure 4, and it is more beneficial to enhance the mechanical properties by introducing fibrous additives than globular ones.

### 3.4. Dynamic Mechanical Analysis

**Figure 8:**
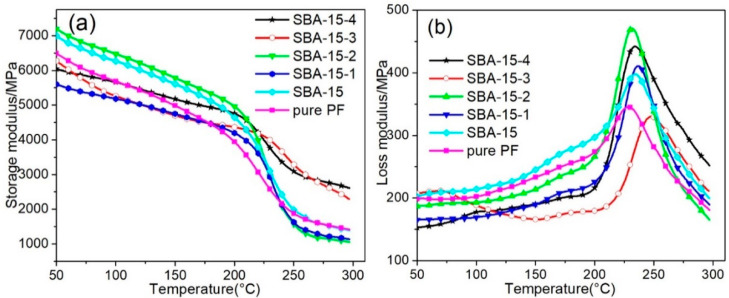
Temperature dependence of the storage modulus (**a**) and loss modulus (**b**) for the SBA-15/PF nanocomposites at 1 Hz frequency.

The dynamic mechanical properties of SBA-15/PF nanocomposites with different pore sizes of SBA-15 were determined within the temperature range from 50 to 300 °C. As shown in Figure 8a, the storage modulus (E′) curves showed a similar trend; firstly, the curves decreased gently in the range of 50–200 °C; then, they decreased sharply between 200 °C and 250 °C, followed by a stable change. Nonetheless, the E′ of the PF nanocomposites was more stable than that of the pure PF composite, especially, the SBA-15-3/PF and SBA-15-4/PF nanocomposites; the E′ values kept on a high level in the range of 250–300 °C. These results could be ascribed to the existence of PF chains or chain segments within the mesoporous channels of SBA-15, which can confine the motions of the PF chains or chain segments; the effect may be more obvious if confined to more polymer chains or chain segments.

Usually, the T_g_ values of polymer composites can be determined by the loss modulus (E″) peak. Figure 8b exhibits the E″-temperature curves for PF nanocomposites with different pore sizes of SBA-15. It is clear that the T_g_ for the PF nanocomposites was higher than for the pure PF composite. Moreover, the SBA-15-3/PF nanocomposite showed the greatest improvement in T_g_ values, which was shifted from 228.0 °C for pure PF to 247.0 °C for SBA-15-3/PF nanocomposites. This result was consistent with the result of DSC, which can demonstrate that the thermomechanical properties of PF nanocomposites could be obviously improved by introducing expanded pores of SBA-15, resulting from the confinement of thermal motion for PF molecule chains.

### 3.5. Friction Properties

**Figure 9:**
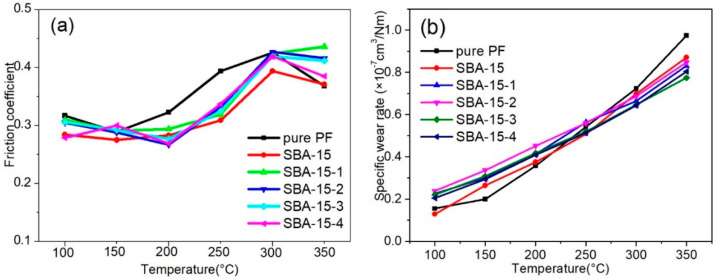
Friction curves of the friction coefficient (**a**) and volume wear rate (**b**) for the SBA-15/PF nanocomposites.

Figure 9 shows the change in the friction coefficient and wear rates of the PF nanocomposites with different pore sizes of SBA-15 as a function of temperature up to 350 °C. As shown in Figure 9a, the variation trend of the friction coefficient was similar. With increasing temperature, the friction coefficients first increased slowly below 200 °C and then, increased rapidly in the range between 200 °C and 300 °C; finally, they decreased slightly when the temperature went up to 350 °C. When the friction disc’s temperature was raised to 300 °C, the friction coefficients of the PF nanocomposites reached a maximum value and then reduced, which were called heat fades due to the decomposition of organic ingredients in the PF nanocomposites [32]. At temperatures below 300 °C, the pure PF composites showed high friction coefficients, indicating that pure PF had a higher bonding strength than the modified SBA-15/PF nanocomposites. Nevertheless, the decline in the friction coefficient for the pure PF composites was the most obvious from 300 °C to 350 °C, expressing that its heat fade was the most apparent. Comparing with the pure PF composite, the SBA-15/PF nanocomposites exhibited a more stable friction coefficient, which could be ascribed to the better thermal stability for the SBA-15/PF nanocomposites modified by the different pore sizes of SBA-15.

The wear rates of the SBA-15/PF nanocomposites with different pore sizes of SBA-15 were measured as a function of temperature and shown in Figure 9b. The wear rates increased gradually in the temperature range between 100 and 350 °C; meanwhile, the wear rates for the SBA-15/PF nanocomposites were higher than the pure PF composite below 200 °C. Their values came close to each other at 250 °C and the wear rates were lower than the pure PF composite in the temperature range of 300–350 °C; specifically, the wear rate was lowest for the SBA-15-3/PF nanocomposite. The results indicated that the wear resistance for the PF nanocomposites can be improved under higher temperatures by introducing different pore sizes of SBA-15. The reasons were the following facts: the wear rates were mainly determined by two factors between bonding strength and thermal stability; and the bonding strength plays a major role at low temperatures. Nevertheless, the effect of thermal stability will be more prominent at high temperatures. Moreover, a part of the PF chains was penetrated through the pore channels of SBA-15, which can not only improve the thermal stability of the PF nanocomposites, but also make the exfoliation of SBA-15 from polymer matrices difficult.

## 4. Conclusions

SBA-15/PF nanocomposites with different pore sizes of mesoporous silica were prepared in this study. SBA-15-n/PF hybrids were initially prepared through in situ polymerization, which were then used as a polymer matrix to fabricate the nanocomposites by compression molding. The SBA-15/PF hybrid illustrated a higher thermal stability than that of pure PF. The DMA results indicated that the **E′** of the nanocomposites enhanced by SBA-15-3 and SBA-15-4 were drastically higher than pure PF at a high temperature, and Tg shifted to higher temperatures as the pore sizes of SBA-15 increased, reaching a maximum value at 247 °C, elevated by 19 °C in comparison with the pure PF composite. The variation of the friction coefficient for the SBA-15/PF nanocomposites was more stable at different temperatures and the wear rate was also lower at a high temperature. The larger pore sizes and pore volumes were conducive to the confinement of the PF chains or chain segments into pore channels, which can, in turn, enhance the thermal and frictional properties of the nanocomposites; in addition, the reinforced matrix material could be used in the fields of heat-resistant materials and friction materials.

## Figures and Tables

**Table 1:** Textural parameters of SBA-15 particles with varying pore size.

Material	BET Surface Area (m^2^/g)	Total Pore Volume (cm^3^/g)	Average Pore Diameter (nm)
SBA-15	637	1.03	7.31
SBA-15-1	664	1.48	11.87
SBA-15-2	721	2.01	17.99
SBA-15-3	731	2.43	19.46
SBA-15-4	719	2.34	20.29

**Table 2:** Thermal decomposition of pure PF and SBA-15/PF enhanced by SBA-15 with different size of mesoporous.

Material	T_d,10,_ °C	T_d,max,_ °C	Char Yield at 800 °C (wt %)
Pure PF SBA-15	327.0 342.2	377.0 385.0	42.1 46.5
SBA-15-1	348.9	392.5	46.9
SBA-15-2	355.7	392.0	46.6
SBA-15-3	340.4	384.0	47.0
SBA-15-4	342.1	390.0	46.8
